# Correction: From atoms to fragments: a coarse representation for efficient and functional protein design

**DOI:** 10.1093/bioinformatics/btag734

**Published:** 2026-10-09

This is a correction to: Leonardo V Castorina, Christopher W Wood, Kartic Subr, From atoms to fragments: a coarse representation for efficient and functional protein design, *Bioinformatics*, Volume 42, Issue 5, May 2026, btag172, https://doi.org/10.1093/bioinformatics/btag172

The following changes have been made to the originally published paper.

The data numbers throughout Table 1 have been emended to be to only two decimal places.

